# Corrigendum: Pharmacological inhibition of adipose triglyceride lipase corrects high-fat diet-induced insulin resistance and hepatosteatosis in mice

**DOI:** 10.1038/ncomms15490

**Published:** 2017-04-25

**Authors:** Martina Schweiger, Matthias Romauch, Renate Schreiber, Gernot F Grabner, Sabrina Hütter, Petra Kotzbeck, Pia Benedikt, Thomas O Eichmann, Sohsuke Yamada, Oskar Knittelfelder, Clemens Diwoky, Carina Doler, Nicole Mayer, Werner De Cecco, Rolf Breinbauer, Robert Zimmermann, Rudolf Zechner

Nature Communications 8: Article number: 14859; DOI: 10.1038/ncomms14859 2017); Published: 03 22 2017; Updated: 04 25 2017

The affiliation details for R. Zimmermann, R. Zechner and R. Breinbauer are incorrect in this Article. The correct affiliation details for these authors are given below:

Institute of Molecular Biosciences, University of Graz, Heinrichstrasse 31, 8010 Graz, Austria.

BioTechMed-Graz, Mozartgasse 12, 8010 Graz, Austria.

