# Supplementary material for: Pharmacological inhibition of adipose triglyceride lipase corrects high-fat diet-induced insulin resistance and hepatosteatosis in mice
Source: Nat Commun. 2017 Mar 22;8:14859. doi: 10.1038/ncomms14859 (PMC5364409; doi:10.1038/ncomms14859)
Supplement: Supplementary Information — Supplementary Figures, Supplementary Table 1. [file ncomms14859-s1.pdf]

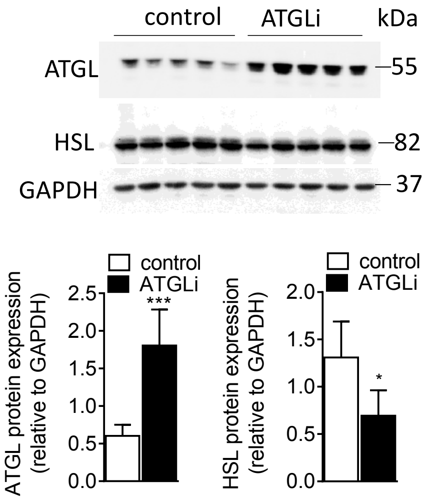

Supplementary Fig. 1 Expression of lipases in WAT of control and ATGLi animals. C57Bl6J mice were fed control HFD or HFD containing Atglistatin for 50 days. 20 µg protein of adipose tissue (gWAT) lysate was subjected to SDS-PAGE and Western Blot analysis using antibodies for ATGL, HSL, and GAPDH and their respective HRP conjugated secondary antibodies. Signal densities were assessed using BioRad Chemidoc™ Software. Data are presented as mean +s.d. Statistical significance was determined by Student's two-tailed *t* test. P < 0.05 (*), P < 0.01 (**), and P < 0.001 (***), n=5

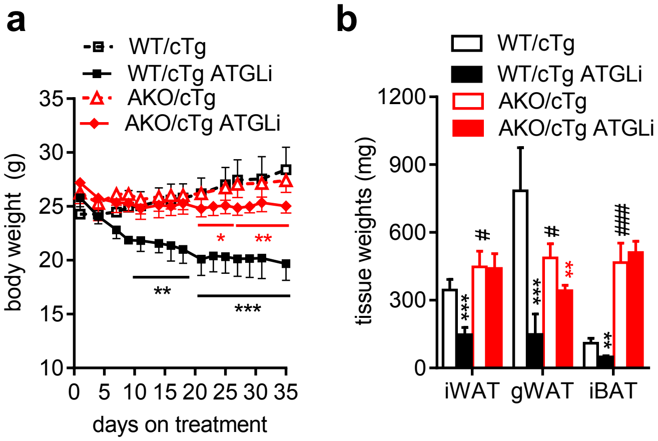

Supplementary Fig. 2. Atglistatin effect in ATGL deficient animals. 8 weeks old ATGL deficient animals expressing ATGL exclusively in the heart (AKO/cTg) or wild type animals containing the heart transgene (WT/cTg) were fed a HFD in the presence or absence of Atglistatin. (a) Body weight was monitored throughout diet intervention. (b) Weights of adipose tissue depots were determined after 35 days of diet intervention. Data represent mean ± s.d. Statistical significance was determined by Student's two-tailed *t* test. For analysis of multiple measurements, we performed one-way analysis of variance (ANOVA) followed by Bonferroni *post hoc* test; P < 0.05 (*), P < 0.01 (**), and P < 0.001 (***) for control vs ATGLi; P < 0.05 ($^{\#}$), P < 0.01 ($^{\#\#}$), and P < 0.001 ($^{\#\#\#}$) for differences between genotypes. n=5-6

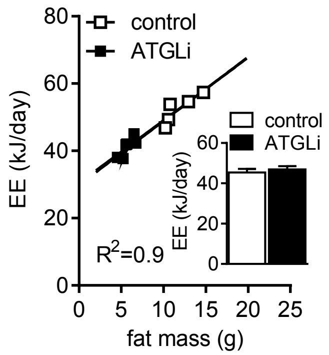

Supplementary Fig. 3. Energy expenditure of ATGLi mice in relation to fat mass. 6 weeks old male C57Bl6J mice were fed a HFD (45 kJ% fat; 22.1 kJ/g) for 50 days. Thereafter, mice were fed a HFD in the presence and absence of Atglistatin for another 50 days. O_2 consumption and CO_2 production were determined using a laboratory animal monitoring system (LabMaster, TSE Systems, n=6 per group). Mice were familiarized with the metabolic cages for 3 days before measurement. Energy expenditure was calculated using the formula: EE(kJ/day)=15,818*VO_2+5,176*VCO_2/1000*24 and is plotted against fat mass. Linear regression analysis was performed using GraphPad prism software. Data represent single mice. (insert) Energy expenditure is expressed as adjusted means based on a normalized body mass of 8.5 g determined using ANCOVA, p=0.64. Data represent mean + s.d.

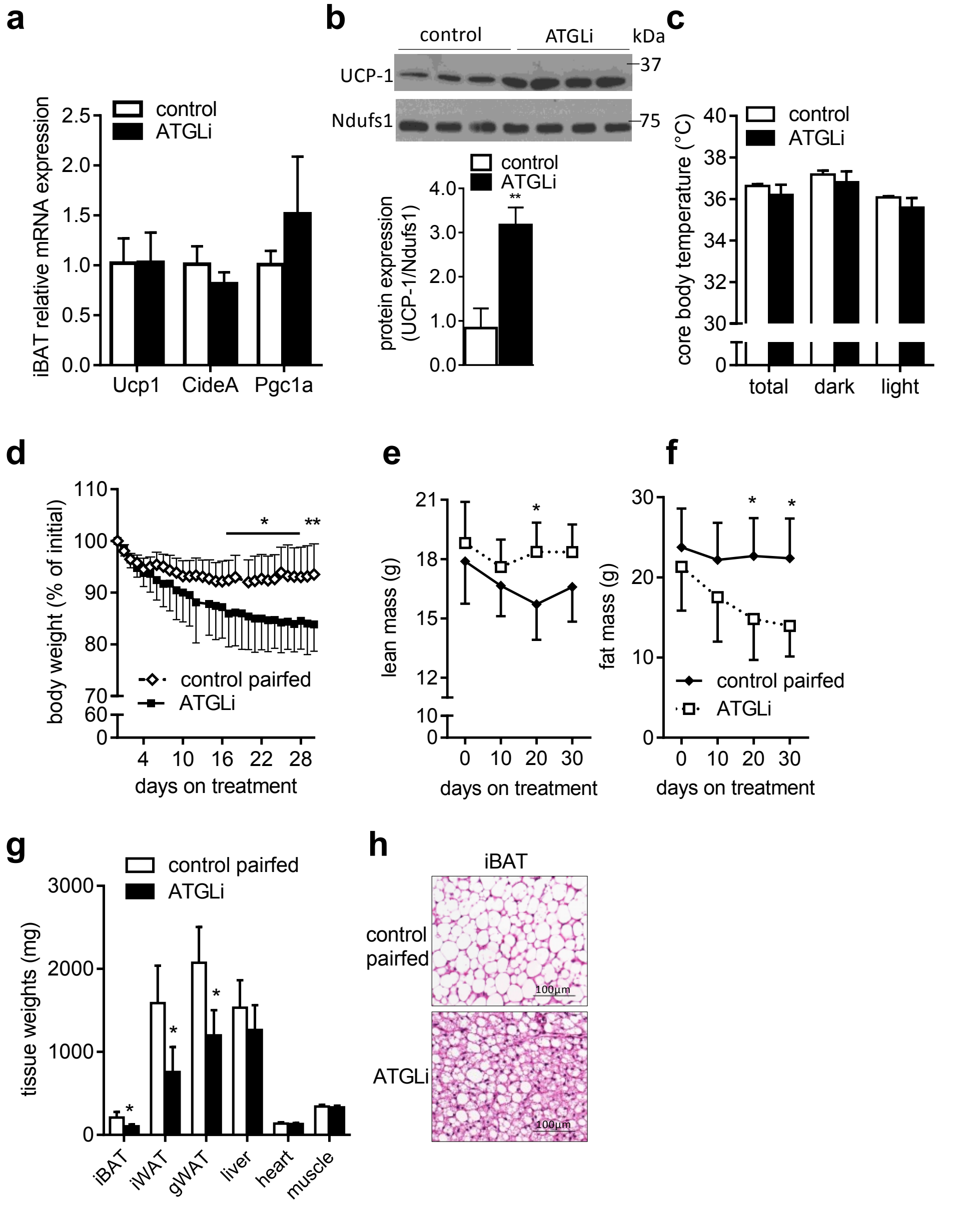

Supplementary Fig. 4. Atglistatin effect on thermogenesis. (a) mRNA expression of Ucp1, CideA, and Pgc1a and (b) protein expression of UCP1 and Ndusf1 was determined in iBAT of 5 h fasted HFD fed control and ATGLi mice after 45 days of treatment. (c) Core body temperature was assessed on 4 consecutive days after 6 and 10 weeks of diet intervention starting 2 weeks after implanting telemetry sensors into the abdominal cavity of mice (n=5 per group). (d-h) Male mice were fed a HFD for 14 weeks at an ambient temperature of 21-23°C and acclimatized to 30°C for further 2 weeks prior to intervention using Atglistatin HFD. The control group was switched to pair-feeding regimen receiving the mean daily food intake of ATGLi group from day 0 onwards. Time-course of (d) relative body weight, (e) total fat mass, and (f) lean mass. (g) Tissue weights of 2 h re-fed animals. (h) Histological images of interscapular BAT (iBAT) of control pair-fed and ATGLi animals (Scale bar 100 µm). Data are presented as mean +/- s.d. Statistical significance was determined using Student's t-test between ATGLi HFD and control HFD ad lib, or ATGLi HFD and control HFD pair-fed. P < 0.05 (*), P < 0.01 (**), and P < 0.001 (***), n = 6-7.

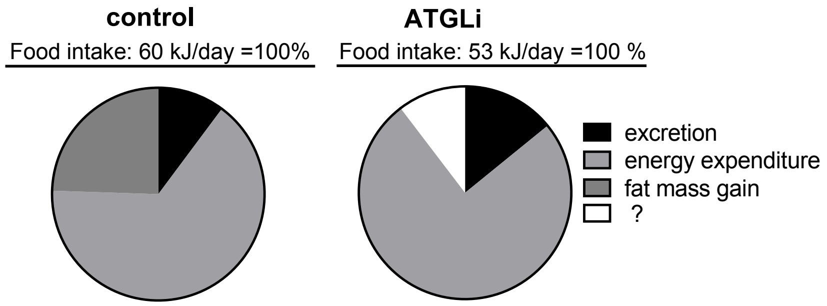

Supplementary Fig. 5. Energy balance of ATGLi animals. Energy balance is plotted as averaged energy intake, fecal excretion, expenditure, and storage (fat mass gain) per day (n=9 per group).

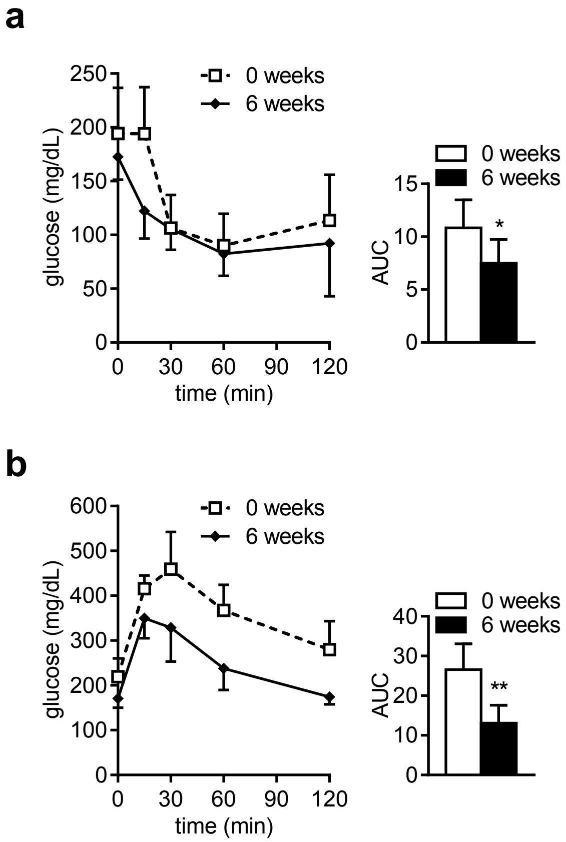

Supplementary Fig. 6. Atglistatin treatment improves glucose homeostasis. 6 weeks old male C57Bl6J mice were fed a HFD (45 kJ% fat; 22.1 kJ/g) for 50 days. Thereafter, mice were fed a HFD in the presence and absence of Atglistatin for 6 weeks. (a)Insulin sensitivity and (b) glucose tolerance was determined after 50 days HFD-feeding before Atglistatin treatment (0 weeks) and 6 weeks after Atglistatin treatment (on a HFD) by intraperitoneal injection of 0.5 IU/kg insulin and 1.5 g/kg glucose, respectively (n=6). Area under the curve (AUC) was calculated using GraphPad Prism software. Data represent mean + s.d. Statistical significance between 0 and 6 weeks was determined by two-tailed student's ttest; *P<0.05; **P<0.01.

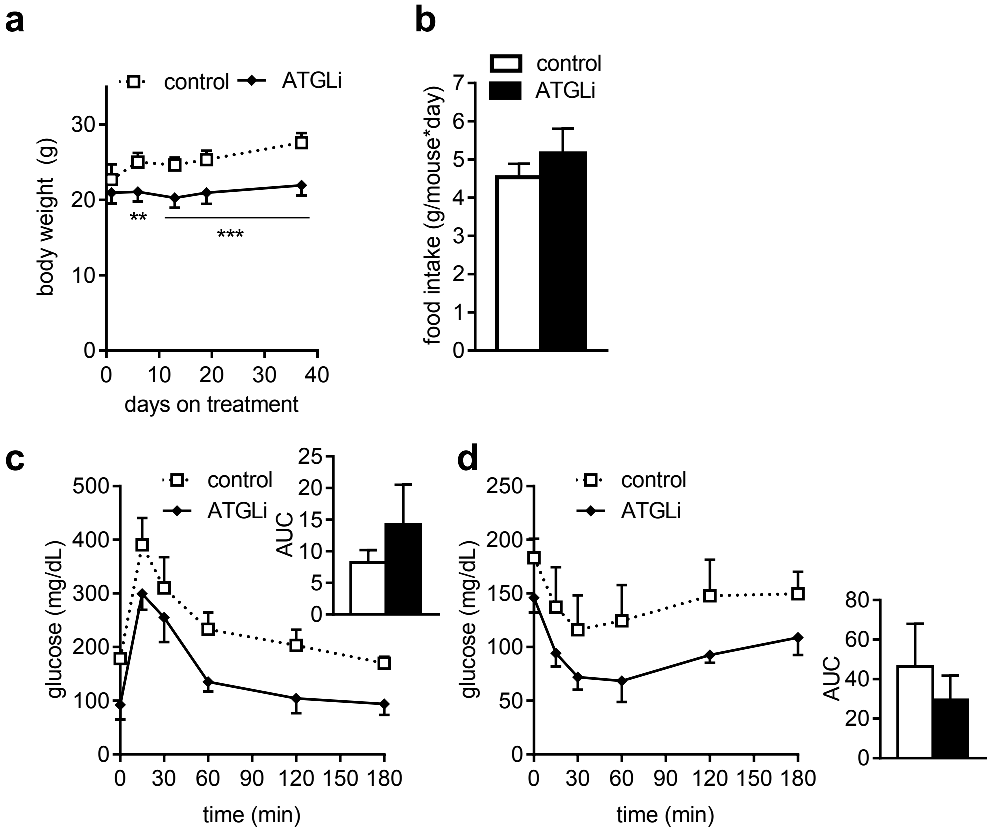

Supplementary Fig. 7. Atglistatin treatment on chow diet. 20 weeks old male C57Bl6J mice were fed a chow diet in the presence of absence of Atglistatin for 40 days. (a) body weight and (b) food intake was monitored regularly. (c) glucose tolerance and (d) insulin sensitivity was determined after 30 and 40 days of diet intervention by intraperitoneal injection of 1.5 g/kg glucose and 0.5 IU/kg insulin, respectively (n=6). Area under the curve (AUC) was calculated using GraphPad Prism software. Data represent mean +/- s.d. Statistical significance between control and ATGLi was determined by two-tailed student's ttest; **P<0.01; ***P<0.001, n=5

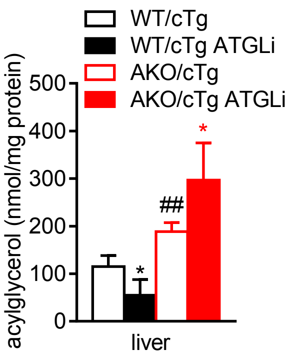

Supplementary Fig.8. Liver TG of Atglistatin treated ATGL deficient animals. 8 weeks old ATGL deficient animals expressing ATGL exclusively in the heart (AKO/cTg) or wild type animals containing the heart transgene (WT/cTg) were fed a HFD in the presence or absence of Atglistatin. Total lipids were extracted and acylglycerol levels were determined in livers of WT/cTg and AKO/cTg mice after 35 days of diet intervention. Data represent mean + s.d. Statistical significance was determined by Student's two-tailed *t* test. For analysis of multiple measurements, we performed one-way analysis of variance (ANOVA) followed by Bonferroni *post hoc* test; $P < 0.05$ (*) for control vs ATGLi; $P < 0.01$ ($^{\#\#}$) for differences between genotypes. n=5-6

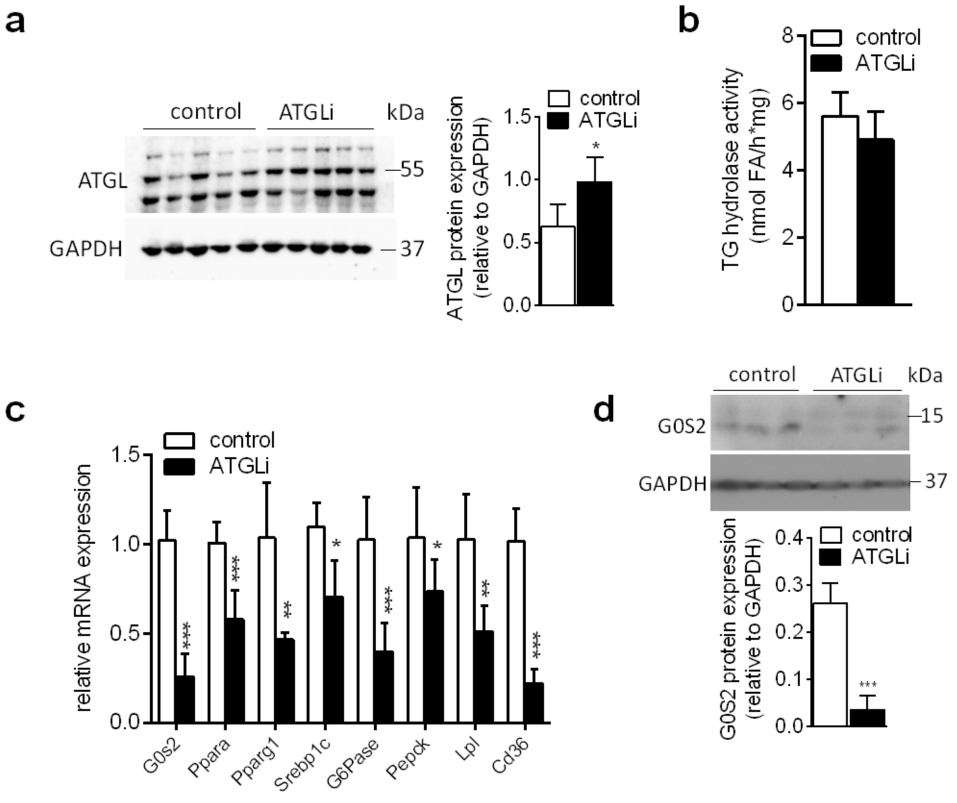

Supplementary Fig. 9. Expression of metabolic genes in livers of ATGLi animals on HFD. 6 weeks old male C57Bl6J mice were fed a HFD (45 kJ% fat; 22.1 kJ/g) for 50 days. Thereafter mice were fed a HFD in the presence and absence of Atglistatin for another 50 days. (a) ATGL and GAPDH protein expression was analyzed by western blot analysis of liver cytoplasmic fractions and quantified relative to GAPDH as housekeeping gene (n=5). (b) TG hydrolase activity of liver lysates from control and ATGLi mice (n=5). (c) mRNA expression of metabolic marker genes was assessed in livers of re-fed animals (n=7 per group). (d) G0S2 protein expression was analyzed by western blot analysis of liver cytoplasmic fractions and quantified relative to GAPDH as housekeeping gene (n=5 per group, 3 representative bands on the western blot). Data represent mean + s.d. Statistical significance was determined by Student's two-tailed *t* test, $P < 0.05$ (*), $P < 0.01$ (**), and $P < 0.001$ (***) for control vs ATGLi.

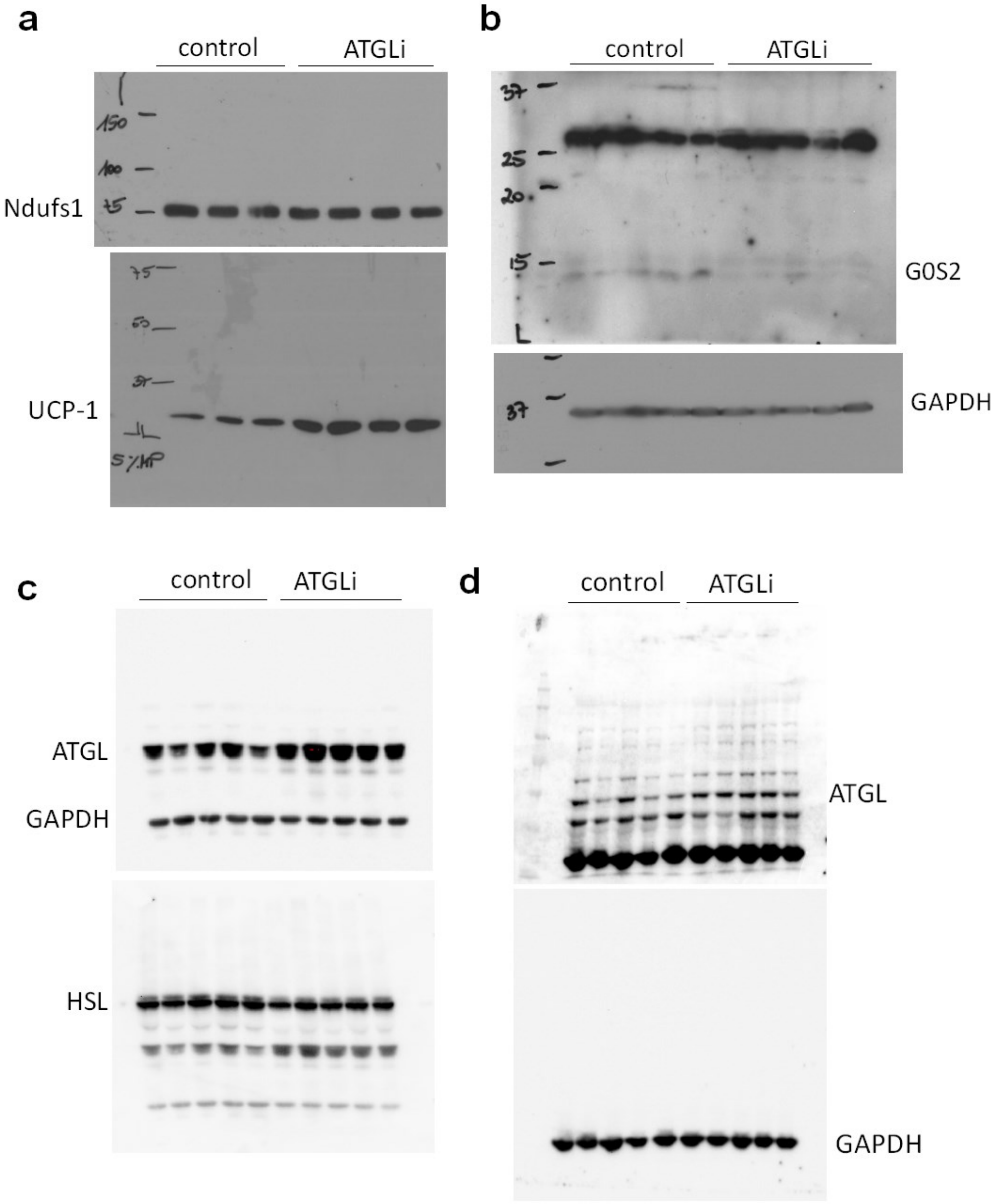

Supplementary Fig. 10. Uncropped scans of Western Blots. (a) UCP-1 and Ndusf1 protein expression in iBAT cytoplasmic fractions. (b) G0S2 and GAPDH protein expression in liver cytoplasmic fractions**.** (c) ATGL, HSL, and GAPDH protein expression in gWAT cytoplasmic fractions. (d) ATGL and GAPDH protein expression in liver cytoplasmic fractions.

Supplementary table 1. Primer sequences used for quantitative gene expression analysis

Gene	Gene synonym	Primer forward	Accession number
glucose-6-phosphatase	G6Pase	5'-CCTCCTCAGCCTATGTCTGC-3'	NM_008061.3
lipoprotein lipase	Lpl	5'-TCCAGCCAGGATGCAACA-3'	NM_008509.2
peroxisome proliferator activated receptor γ 1	Pparγ1	5′-AACAAGACTACCCTTTACTGAAAT-TACCA-3′	NM_001127330.1
sterol regulatory element binding transcription factor 1	Srebp1c	5′-GTTACTCGAGCCTGCCTTCAGG-3′	NM_011480.3
phosphoenolpyruvate carboxykinase 1	Pepck	5′-CATATGCTGATCCTGGGCATAAC-3′	NM_011044.2
fatty acid translocase	Cd36	5′-GAACCTATTGAAGGCTTACATCC-3′	NM_001159555.1
diacylglycerol O-acyltransferase 2	Dgat2	5′-TTCCTGGCATAAGGCCCTATT-3′	NM_026384.3
ribosomal protein large, P0	36B4	5′-GCTTCATTGTGGGAGCAGACA-3′	NM_007475.5
adhesion G protein-coupled receptor E1	F4/80	5′-GGATGTACAGATGGGGGATG-3′	NM_010130.4
integrin alpha X	Cd11c	5′-CAGTGACCCCGATCACTCTT-3′	NM_021334.2
interleukin 6	IL-6	5'-GAGGATACCACTCCCAACAGACC-3'	NM_031168.1
Transforming growth factor, β 1	Tgfβ	5'-CACCGGAGAGCCCTGGATA-3'	NM_011577.1
tumornecrosis factor- α	Tnfα	5′-GACCCTCACACTCAGATCATCTTCT-3‘	NM_013693.3
Interleukin 1 β	IL1β	5'-CACAGCAGCACATCAACAAG-3'	NM_008361.3
Collagen type I alpha 2	Col1a2	5'-AAGGGTGCTACTGGACTCCC-3'	NM_007743.2
Collagen type I alpha 1	Col1a1	5′-CCGGCTCCTGCTCCTCCTA-3	NM_007742.3
Peroxisome proliferator activated receptor α	Pparα	5′-GTACCACTACGGAGTTCACGCAT-3'	NM_001113418.1
G0/G1 switch gene 2	G0s2	5'-TAGTGAAGCTATACGTGCTGGGC-3'	NM_008059.3
Peroxisome proliferative activated receptor, gamma, coactivator 1 α	Pgc1α	5'-CCCTGCCATTGTTAAGACC-3'	NM_008904
Carnitine palmitoyltransferase 1 β	Cpt1β	5'-CGAGGATTCTCTGGAACTGC-3'	NM_009948
Uncoupling protein 1	Ucp1	5'-ACTGCCACACCTCCAGTCATT -3'	NM_009463.3
cell death-inducing DNA fragmentation factor α	CideA	5'- TGCTCTTCTGTATCGCCCAGT -3'	NM_007702.2
Angiopoietin like protein 4	Angptl4	5'-GTTTGCAGACTCAGCTCAAGG-3'	NM_020581.2
Fatty acid binding protein 4	Ap2	5'-AAGGTGAAGAGCATCATAACCCT-3'	NM_024406.2
